# Evolutionarily new genes in humans with disease phenotypes reveal functional enrichment patterns shaped by adaptive innovation and sexual selection

**DOI:** 10.1101/2023.11.14.567139

**Published:** 2024-04-22

**Authors:** Jianhai Chen, Patrick Landback, Deanna Arsala, Alexander Guzzetta, Shengqian Xia, Jared Atlas, Dylan Sosa, Yong E. Zhang, Jingqiu Cheng, Bairong Shen, Manyuan Long

**Affiliations:** 1 Department of Ecology and Evolution, The University of Chicago, 1101 E 57th Street, Chicago, IL 60637; 2 Institutes for Systems Genetics, West China University Hospital, Chengdu 610041, China; 3 Department of Pathology, The University of Chicago, 1101 E 57th Street, Chicago, IL 60637; 4 Key Laboratory of Zoological Systematics and Evolution, Institute of Zoology, Chinese Academy of Sciences, Beijing 100101, China

**Keywords:** New genes, Pleiotropy, Young genes, Phenotypic innovation, Sexual selection, Natural selection

## Abstract

New genes (or young genes) are genetic novelties pivotal in mammalian evolution. Their phenotypic impacts and evolutionary pattern over time, however, remain elusive in humans due to the technical and ethical complexities in functional studies. By combining human gene age dating and Mendelian disease phenotyping, our research reveals a gradual increase in disease gene proportions with gene age. Logistic regression modeling indicates that this increase could be related to longer protein lengths and higher burdens of deleterious de novo germline variants (DNVs) for older genes. We also find a steady integration of new genes with biomedical phenotypes into the human genome over macroevolutionary timescales (~0.07% per million years). Despite this stable pace, we observe distinct patterns in phenotypic enrichment, pleiotropy, and selective pressures across gene ages. Notably, young genes show significant enrichment in diseases related to the male reproductive system, indicating strong sexual selection. Young genes also exhibit disease-related functions in tissues and systems potentially linked to human phenotypic innovations, such as increased brain size, musculoskeletal phenotypes, and color vision. We further reveal a logistic growth pattern of pleiotropy over evolutionary time, indicating a diminishing marginal growth of new functions for older genes due to intensifying selective constraints over time. We propose a “pleiotropy-barrier” model that delineates higher potentials of phenotypic innovation for young genes than for older genes, a process subject to natural selection. Our study demonstrates that evolutionary new genes are critical in influencing human reproductive evolution and adaptive phenotypic innovations driven by sexual and natural selection, with low pleiotropy as a selective advantage.

## Introduction

The imperfection of DNA replication serves as a rich source of variation for evolution and biodiversity (Clausius 1879; and and Bebenek 2000; Vanchurin et al. 2022). Such genetic variations underpin the ongoing evolution of human phenotypes, with beneficial mutations being fixed by positive selection, and detrimental ones being eliminated through purifying selection. In medical terminology, this spectrum is categorized as “case and control” or “disease and health,” representing two ends of the phenotypic continuum (Pavličev and Wagner 2022). Approximately 8,000 clinical types of rare Mendelian disorders, affecting millions worldwide, are attributed to deleterious DNA mutations in single genes (monogenic) or a small number of genes (oligogenic) with significant effects (Antonarakis and Beckmann 2006; Fetro and Scherman 2020). To date, over 4,000 Mendelian disease genes have been identified, each contributing to a diverse array of human phenotypes (https://mirror.omim.org/statistics/geneMap) (Boycott et al. 2013). These identified disease genes and associated phenotypes could provide critical insights into the evolutionary trajectory of human traits (Claussnitzer et al. 2020).

Evolutionarily new genes – such as *de novo* genes, chimeric genes, and gene duplicates – have been continually emerging and integrating into the human genome throughout the macroevolutionary process of human lineage (Brosius 1991; Kaessmann et al. 2002; Conrad and Antonarakis 2007; Baertsch et al. 2008; Wu et al. 2011; Long et al. 2013; Van Oss and Carvunis 2019; Betrán and Long 2022; Zhang et al. 2022). Decades of genetic studies in non-human systems have provided extensive evidence contradicting this intuitive argument. New genes can integrate into biologically critical processes, such as transcription regulation, RNA synthesis, and DNA repair (Ciccarelli et al. 2005; Ding et al. 2021). For instance, in yeast, some *de novo* genes play roles in DNA repair process (Cai et al. 2008; Li et al. 2010; Parikh et al. 2022). In *Drosophila* species, lineage-specific genes can control the key cytological process of mitosis (Ross et al. 2013). New genes have also been found with roles in early larval development of *Drosophila* (Kasinathan et al. 2020). In nematodes, insects, and fish, some lineage-specific genes are thought to be involved in the functions of morphological development, a process which was long believed to be governed by deeply conserved genetic mechanisms (Ragsdale et al. 2013; Klomp et al. 2015; Li et al. 2021). These studies from model species reveal various important biological functions of new genes.

Compared to non-human model organisms, where gene functions can be characterized through genetic knockdowns and knockouts, interrogating functions of human genes in their native context is unfeasible. Despite this limitation, numerous omics data and *in vitro* studies in human genes have suggested the potential roles of evolutionary young genes in basic cellular processes and complex phenotypic innovations (Marques et al. 2005; Shi and Su 2012; Zhang and Long 2014). Brain transcriptomic analysis has revealed that primate-specific genes are enriched among up-regulated genes early in human development, particularly within the human-specific prefrontal cortex (Zhang et al. 2011). The recruitment of new genes into the transcriptome suggests that human anatomical novelties may evolve through the contribution of new gene evolution. Recent studies based on organoid modeling also support the importance of *de novo* genes on human brain size enlargement (An et al. 2023; Rich and Carvunis 2023). These lines of evidence in recent decades about the functions of new genes contradict the conventional conservation-dominant understanding of human genetics and phenotypes.

It has long been found that there are more disease genes in older genes than in young ones (Domazet-Lošo and Tautz 2008). However, the underlying mechanism remains unclear. In recent years, increasing availability of large-scale sequencing of whole exomes and whole genomes have greatly promoted the identification of deleterious variants underlying rare disorders (Richards et al. 2015). Statistical and medical studies have demonstrated that rare diseases are often caused by rare variants, which have more significant effects on disease phenotypes than common variants (Richards et al. 2015; Wang et al. 2021; Chen et al. 2022; Greene et al. 2023; Jia et al. 2023; Weiner et al. 2023; Chen et al. 2024a). The effect of gene-based rare variant burden—the aggregate impact of rare (including de novo germline) protein-altering variants—has been confirmed in many genetic disorders (Purcell et al. 2014; Zuk et al. 2014; Guo et al. 2018; Halvorsen et al. 2020; Jiang et al. 2021).

In this study, we tackled the complexities of human phenotypic evolution and the underlying genetic basis by integrating gene age dating with analyses of Mendelian disease phenotypes. As a direct indicator of functional effects, the anatomical organ/tissue/system phenotypes (OP) affected by causal genic defects allow us to understand the influence of gene ages on phenotypic enrichment, pleiotropy, and selective constraints along evolutionary journey. We revealed higher gene-wise deleterious DNVs burden in older genes than in young genes, suggesting higher susceptibility of older genes to genetic diseases. This finding is also consistent with stronger purifying selection in older genes to remove deleterious mutations. Notably, disease gene emergence rates per million years were found to be similar among different macroevolutionary stages, suggesting the continuous integration of young genes into biomedically important phenotypes. Despite the stable pace of gene integration per million years, young genes impact limited number of disease systems, with an enrichment in the male reproductive system and the nervous system, suggesting their lower pleiotropy and accelerated sexual selection and human-specific adaptive innovation. By contrast, older genes are higher in pleiotropic burden that impacts more anatomical systems and are under stronger purifying selection (selective constraints). These patterns suggest that the gene age as an important factor to influence levels of pleiotropy. New genes can rapidly become the genetic bases of human critical phenotypes, especially the reproductive and innovative traits, a process likely facilitated by their low pleiotropy.

## Results

### Ages and organ/tissue phenotypes of human genetic disease genes

**Figure 1.**
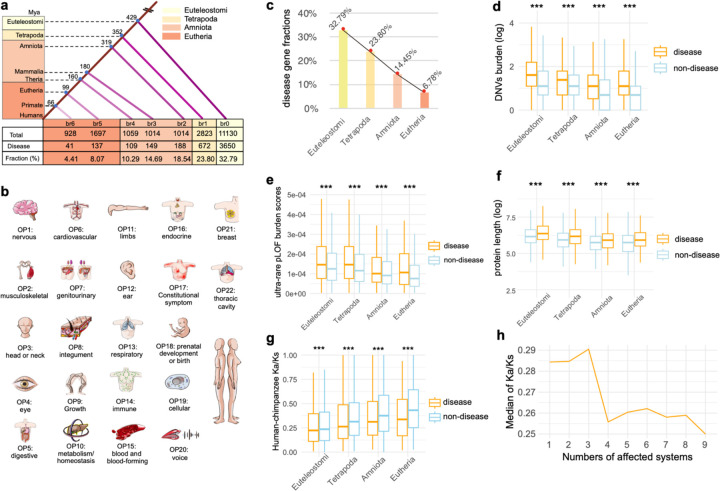
Number distribution and Ka/Ks ratios of genes categorized by ages and disease phenotypes (also organ phenotype genes). (**a**) The phylogenetic framework illustrating gene ages and disease genes associated with organ phenotypes. The phylogenetic branches represent age assignment for all genes and disease genes. The “br” values from br0 to br6 indicate different age groups (or branches). These are further categorized into four evolutionary age stages. The vertical axis depicts the divergence time sourced from the Timetree database (July 2023). The numbers of total genes and disease genes and their ratios are shown for each evolutionary age stage. (**b**) The 22 HPO-defined organ/tissue systems, which are ordered based on the proportion of genes among all disease genes. (**c**) The proportions of disease genes at four major evolutionary age groups from Euteleostomi to Eutheria. (**d**) The gene-wise burden based on de novo germline variants from Gene4Denovo database (Zhao et al. 2020) between disease and non-disease genes across four major evolutionary age groups. (**e**) The burden score of ultrarare predicted loss-of-function (pLOF) variants (Weiner et al. 2023) between disease and non-disease genes across four major evolutionary age groups. (**f**) The protein lengths between disease and non-disease genes across four major evolutionary age groups. (**g**) The pairwise Ka/Ks ratios from Ensembl database based on Maximum Likelihood estimation for “one to one” orthologs between human and chimpanzee. Only genes under purifying selection are shown (Ka/Ks < 1). (**h**) The line plot for the number of affected systems for disease genes and their pairwise Ka/Ks ratios for “one to one” orthologs between human and chimpanzee. Only genes under purifying selection are shown (Ka/Ks < 1). Note: all significance levels are determined using the Wilcoxon rank sum test, comparing disease genes to non-disease genes. The symbol “***” indicates significance level of *p* < 0.001.

We determined the ages of 19,665 non-redundant genes, following the phylogenetic framework of the GenTree database (Shao et al. 2019) and gene model annotations from Ensembl v110 (Supplementary Table 1). To ensure sufficient power in statistical comparisons across, we merged evolutionary age groups with a small number of genes (<100) into their adjacent older group (Figure 1a). As a result, we reclassified these genes into seven ancestral age groups, ranging from Euteleostomi (or more ancient) nodes to modern humans (br0-br6, Figure 1a). These evolutionary groups have been further categorized into four evolutionary age epochs, starting from the oldest, Euteleostomi, to progressively younger stages of Tetrapoda, Amniota, and Eutheria, each containing over 2000 genes. Disease gene data were sourced from the Human Phenotype Ontology database (HPO, Sep 2023), which is the *de facto* standard for phenotyping of rare Mendelian rare diseases (Köhler et al. 2018). This repository synthesizes information from diverse databases, including Orphanet (INSERM 1997; Weinreich et al. 2008), DECIPHER (Wright 2015), and OMIM (Hamosh et al. 2000). An intersection of these data sets yielded 4,946 genes annotated with both evolutionary age and organ/tissue/system-specific phenotypic abnormalities (Figure 1a-1b and Supplementary Table 2).

We observed an increase in the proportion of disease genes over evolutionary time (Figure 1a and 1c), suggesting that gene age impacts disease susceptibility—a trend consistent with earlier studies (Domazet-Lošo and Tautz 2008). Recent advances in medical genetics have revealed that Mendelian disorders are predominantly influenced by rare variants due to their significant phenotypic effects, a rational underlying the widespread use of burden tests in the field (Lee et al. 2014; Guo et al. 2018; Kingdom et al. 2024). However, the correlation between gene age and rare variant burden remains elusive. In addition, assuming a random mutation process, the rare variant burden could be a function of protein length. We analyzed the relationship between rare variant burdens, gene age, and protein length, using data from predicted deleterious de novo germline mutations (DNVs) from 46,612 trios (Wang et al. 2022) and rare variants from 76,215 genomes in the gnomAD database (v4.0.0, minor allele frequency (MAF) < 0.0001) (Chen et al. 2024b). Our findings showed strong positive correlations: protein length with DNVs burden (coefficient = 0.57, *p* < 2.2e-16) and rare variant burden (coefficient = 0.89, *p* < 2.2e-16). Additionally, gene age (mya) also showed a positive correlation with DNV burden (Pearson’s r = 0.13, *p* < 2.2e-16) and rare variant burden (Pearson’s r = 0.14, *p* < 2.2e-16). When using different methods for human gene age dating—gene-family based (Neme and Tautz 2013) and synteny-based method (Shao et al. 2019)—alongside different datasets on de novo variant (DNV) burdens (Zhao et al. 2020), we also found the significant correlation between gene age and DNVs burden (Supplementary Figure 1). These correlations could not be inflated by mutation rate difference among genes, as no significant correlation was observed between gene age (*T, mya*) and DNVs per nucleotide (Pearson’s product-moment correlation, *p* = 0.17).

The role of variant burdens on disease genes was analyzed based on datasets of gene-wise DNVs burden of 68,404 individuals from the Gene4Denovo database (Zhao et al. 2020) and the ultrarare predicted loss-of-function variant (pLoF, MAF < 1 × 10^−5^) burden from 394,783 exomes (Weiner et al. 2023). We observed that disease genes consistently exhibit a higher burden across all major evolutionary age groups (Wilcoxon rank sum test, *p* < 2.2e-16, Figure 1d-1e). Disease genes consistently exhibited longer proteins compared to non-disease genes across all major age groups (Figure 1f). To understand quantitative roles of these factors, we assigned binary states (“1”, disease genes; “0”, non-disease genes) to all genes and performed stratified Logistic regression modeling (Supplementary Table 4). We explored multiple predictors including gene age (*T, mya*), gene length (*L*_*g*_) or protein length (*L*), DNVs burden (*D*) (Wang et al. 2022), and rare variant burden (R) based on gnomAD genomes (Chen et al. 2024b) (Supplementary Table 3). Model comparison was conducted based on likelihood ratio test (LRT) and Akaike information criterion (AIC) (Supplementary Table 4). The optimal model (M9) revealed the significant effects of three significant variables (DNV burden, gene age, and protein length), where the likelihood of being a disease gene increases with protein length (*L* at logarithm scale, coefficient = 0.15; *p* < 0.044), DNVs burden (*D*, coefficient = 0.09; *p* < 8.9e-07), and gene age (*T,* coefficient = 0.005; *p* < 2e-16) (Table 1). The Variance Inflation Factor (VIF) values for these three variables ranged from 1.02 to 1.72, well below the multicollinearity concern thresholds of 5 to 10, suggesting minimal impact on our model.

### Purifying selection intensifies with gene age and is stronger in disease genes than in non-disease genes.

To understand if disease genes evolve under different evolutionary pressures compared to non-disease genes, we compared the metric of Ka/Ks ratio, which is the ratio of the number of nonsynonymous substitutions per nonsynonymous site (Ka) to the number of synonymous substitutions per synonymous site (Ks). Values of Ka/Ks ratio less than 1 suggest the degree of evolutionary constraint (acting against change) (Yang and Bielawski 2000). We retrieved the “one to one” human-chimpanzee orthologous genes and the corresponding pairwise Ka/Ks ratios (12830 genes) from Ensembl database. We also evaluated whether the pattern is consistent with Ka/Ks ratios of human-bonobo and human-macaque orthologs. To include more orthologous genes, we did not use Ka/Ks ratios based on more distant species (such as the test of branch-model). Interestingly, Ka/Ks ratios were consistently lower in disease genes than in non-disease genes for human-chimpanzee orthologs (0.250 vs. 0.321), human-bonobo orthologs (0.273 vs. 0.340), and human-macaque orthologs (0.161 vs. 0.213) (Wilcoxon rank sum test, *p* < 2.2e-16 for all three datasets). These results revealed that disease genes are under significantly stronger purifying selection than non-disease genes, suggesting the important component of selective pressure in constraining the sequence evolution of disease genes. In addition, we observed an increase in Ka/Ks ratios (< 1) for genes from older to younger stages, suggesting a trend of relaxed purifying selection in young genes (Figure 1g and Supplementary Figure 2), which is consistent with some previous studies (Carvunis et al. 2012; Prabh et al. 2018; Montañés et al. 2023). Notably, despite the relaxation of purifying selection for younger genes, disease genes still tend to show lower Ka/Ks ratio than non-disease genes, suggesting a general pattern of stronger purifying selection in disease genes during evolutionary process.

We observed a heterogeneous distribution of disease genes underlying 22 HPO-defined anatomical systems, suggesting varied genetic complexity for diseases of different systems (Supplementary Figure 3a). None of disease genes were found to impact all 22 systems. In contrast, 6.96% of disease genes (344/4946) were specific to a single system’s abnormality. Notably, four systems – the genitourinary system (with 81 genes), the eyes (68 genes), the ears (63 genes), and the nervous system (55 genes) – collectively represented 77.62% of these system-specific genes (267/344, Supplementary table 2). The nervous system displayed the highest fraction of diseases genes (79%, Supplementary Figure 3b). A significant 93.04% of genes were linked to the abnormalities of at least two systems (4602/4946), indicating broad disease impacts or pleiotropy for human disease genes on multiple anatomical systems. This phenotypic effect across systems might arise from the complex clinical symptoms of rare diseases that manifests in multiple organs, tissues, or systems, which could indicate the levels of pleiotropy (Hodgkin 1998; Paul 2000; Lobo 2008). Hence, the comprehensive and deep phenotyping offered by HPO delivers a more systematic perspective on the functional roles of human disease genes, compared to the commonly used functional inferences based on human gene expression profile or *in vitro* screening. Interestingly, we discovered a significant negative correlation between the median Ka/Ks ratios and the number of affected anatomical systems in disease genes (Pearson correlation coefficient = −0.83, *p* = 0.0053). This implies that disease genes with higher pleiotropy, which impact multiple anatomical systems, face more stringent evolutionary constraints compared to genes with lower pleiotropy (Figure 1h).

### Disease gene emergence rate per million years is similar across macroevolutionary epochs.

**Figure 2.**
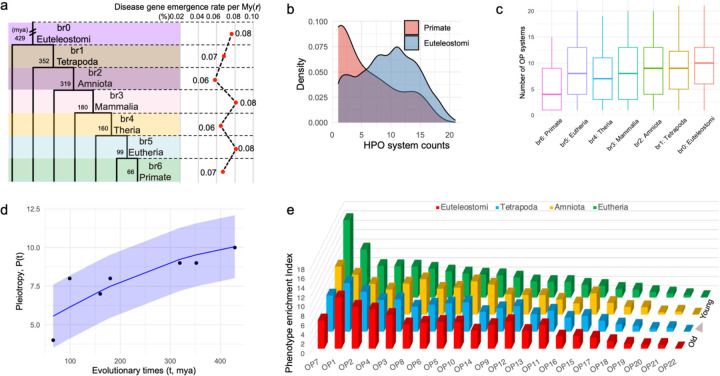
Disease gene emergence rates, phenotypic system coverage, and disease phenotype enrichment index (PEI) along evolutionary age groups. (**a**) The disease-gene emergence rate per million years (*r*) across evolutionary epochs. (**b**) Density distributions showcase the numbers of affected organ phenotypic systems (OPs) for genes originated at primate and Euteleostomi stage. (**c**) Boxplot distributions showcase the numbers of affected organ phenotypic systems (OPs) for genes grouped by their evolutionary age (median values are 4, 8, 7, 8, 9, 9, 10, from left to right). (**d**) The nonlinear least squares (NLS) regression between pleiotropy score (P) and evolutionary times *t* with the logistic growth function ($\text{P}\left(t\right)=\frac{P\_max}{1+\frac{P-max-P\_0}{P\_0}e^{-k\left(t\right)}}$, *k* = 1.66, *p* = 0.000787. The 95% confidence interval is shown shade. P_max and P_0 are empirical medians 10 and 4, respectively) (**e**) The distribution of age and phenotype for the phenotype enrichment index (PEI). The bar plots, colored differently, represent various age epochs, namely Euteleostomi, Tetrapoda, Amniota, and Eutheria, in ascending order of age. The organ phenotypes (OP) are displayed on the horizontal axis and defined in Figure 1c. The standard deviation*s* of PEI are 3.67 for Eutherian epochs and approximately 2.79 for older epochs.

To comprehend whether different evolutionary epochs have different emergence rate for disease genes, we assessed the disease gene emergence rate per million years across macroevolutionary stages from Euteleostomi to Primate (*μ*_*da*_). Considering the sampling space variations at different age group, we calculated *μ*_*da*_ as the fraction of disease genes per million years at each stage (Figure 2a). Although the proportions of disease genes were found to gradually increase from young to old age groups (Figure 1a), the rate *μ*_*da*_ is nearly constant ~0.07% per million years for different age groups (Figure 2a). This constant disease gene emergence rate suggests a continuous and similar fraction of genes evolving to have significant impacts on health, for genes arising in lineages from Euteleostomi to Primate.

Using the recently reported average human generation time of 26.9 years (Wang et al. 2023), the most updated number of coding genes (19,831 based on Ensembl v110), and assuming the simplified monogenic model (Richards et al. 2015), we estimated the number of casual genes for rare diseases per individual per generation (*μ*_*d*_) as 3.73 × 10^−4^ (= 19,831 × 26.9 × 0.07 × 10^−8^). Using this rate, we can derive the rare disease prevalence rate (*r*_*RD*_ = 10,000 x *μ*_*d*_), which equates to approximately 4 in 10,000 individuals. This prevalence agrees remarkably well with the EU definition of rare disease rate prevalence of 5 in 10,000 people (Stolk et al. 2006). The constant parameter highlights the idea that young genes continually acquire functions vital for human health, which agrees with previous observations of young genes and their importance in contributing to phenotypic innovations (Kaessmann 2010; Chen et al. 2013; Xia et al. 2021).

### Pleiotropy growth rate is faster in younger genes following a logistic growth pattern.

Despite the nearly constant integration of young genes into crucial biological functions (Figure 2a), it remains uncertain if gene age could influence disease phenotypic spectrums (or pleiotropy). The overall distribution of OP system counts for disease genes (Supplementary Figure 3a) is similar with the distribution of gene expression breath across tissues (Supplementary Figure 4a-4c). The distribution for the numbers of OP systems showed that young genes have lower peak and median values than older genes (Figure 2b-2c). This pattern was consistent with the results that younger genes tend to express in a limited range of tissues, while older genes exhibit a broader expression profile (Supplementary figure 4d), which also aligns with previously reported expression profiles (Zhang et al. 2012; Long et al. 2013; Carelli et al. 2016; Miller et al. 2022). We found an increasing trend for the median numbers of OP systems from young to old evolutionary epochs (Figure 2c). Interestingly, the increase rates $\left(\frac{\Delta OP\_median}{\Delta t}\right)$ are higher at the younger epochs than other older ones (0.12/mya at Eutherian stage vs. 0.05/mya at older stages on average, Supplementary table 4a), suggesting a non-linear and restricted growth model for the level of pleiotropy over time. We applied a logistic growth function and observed a significant pattern: as evolutionary time increases, the level of pleiotropy rises (Figure 2d, *p* < 0.001). Moreover, the logistic model demonstrates a diminishing marginal growth for pleiotropy over time, indicating that the rate of increase in pleiotropy slows down over evolutionary time. This pattern suggests that pleiotropy is initially lower in new genes but increases at a faster rate compared to older genes. This result is also consistent with the finding that purifying selection gradually increase over evolutionary time from young to old (Figure 1g), which could limit the space of pleiotropy growth.

### Young genes are highly enriched into the reproductive and nervous system diseases.

To understand the enrichment pattern of disease phenotypes for young and old genes, we introduced a metric of the disease phenotype enrichment index (PEI), which quantifies the range of phenotypes across multiple systems (see method for details). Our findings revealed that the most ancient genes, specifically from the Euteleostomi and Tetrapoda periods, had the strongest PEI association with the nervous system (OP1). Conversely, young genes from Amniota and Eutheria epochs tend to display the highest PEI for disease phenotypes of the genitourinary system (OP7) and the nervous system (OP1), with OP7 showing a 38.65% higher PEI than OP1 (Figure 2e, Supplementary Table 4). Among the 22 disease phenotype systems, only the reproductive system (OP7) was unique in showing a steady rise in PEI from older epochs to younger ones (Figure 2e). There were smaller variations in PEI for the older epochs when compared to the more recent Eutheria epoch (~2.79 vs. 3.67), hinting that older disease genes impact a greater number of organ systems, as also shown in Figure 2c. This finding is consistent with the “out-of-testis” hypothesis (Kaessmann 2010), which was built on many observations where the expression patterns of young genes are limited to the testes and can have vital roles in male reproduction. As genes evolve, their expression patterns tend to broaden, potentially leading to phenotypic effects that impact multiple organ systems.

Apart from the reproductive system (OP7), we found that the nervous system (OP1) showed the second highest PEI for Eutherian young disease genes (Figure 2e). Moreover, 42% of the 19 Primate-specific disease genes with diseases affecting the nervous system (OP1) correlate with phenotypes involving brain size or intellectual development (*CFC1*, *DDX11*, *H4C5*, *NOTCH2NLC*, *NOTCH2NLA*, *NPAP1*, *RRP7A*, and *SMPD4*. Supplementary Table 2 and Discussion), consistent with the expectation of previous studies based on gene expression (Zhang et al. 2011). Furthermore, young genes emerging during the primate stage are connected to disease phenotypic enrichment in other adaptive systems, particularly in the HPO systems of the head, neck, eyes, and musculoskeletal structure (Figure 2e). Overall, the Primate-specific disease genes could impact phenotypes from both reproductive and non-reproductive systems, particularly the genitourinary, nervous, and musculoskeletal systems (Supplementary Table 2), supporting their roles in both sexual and adaptive evolution.

### Sex chromosomes are enriched for male-reproductive disease genes: the male hemizygous effect.

**Figure 3.**
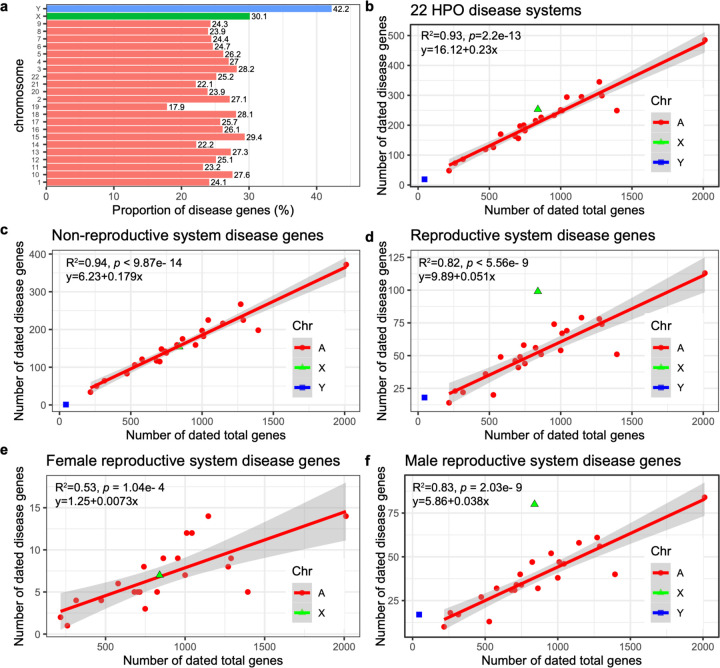
(**a**) The proportions of disease genes across chromosomes. The pink bars represent the autosomes, while green and blue indicate the X and Y chromosomes, respectively. The proportions (%) for different chromosomes are shown above bars. (**b**) The linear regression plotting of disease gene counts against the numbers of total genes with age information on chromosomes. (**c**) Numbers of genes related to the abnormality of non-genitourinary system (non-reproductive system) are plotted against all protein-coding genes on chromosomes with gene age information. (**d**) Numbers of genes related to the abnormality of genitourinary system (the reproductive system) are plotted against all protein-coding genes on chromosomes with gene age information. (**e**) Linear regression of dated disease gene counts against the total numbers of dated genes on chromosomes for female-specific reproductive disease genes. (**f**) Linear regression of dated disease gene counts against the total numbers of dated gene on chromosomes for male-specific reproductive disease genes. The autosomal linear models are displayed on the top left corner. Note: All linear regression formulas and statistics pertain only to autosomes. “A”, “X”, and “Y” indicate autosomes, X and Y chromosomes, respectively.

Considering the rapid concentration of the youngest disease genes in the reproductive system (Figure 2e, OP7), we hypothesized that disease genes could have skewed chromosomal distributions. First, we examined the distribution of all disease genes and found a distinct, uneven spread across chromosomes (Figure 3a and Supplementary Table 4). The X and Y chromosomes contain a higher number of disease genes compared to the autosomal chromosomes. While autosomes have a linear slope of 0.23 (Figure 3b, *R*^2^ = 0.93; *p* = 2.2 × 10^−13^), the Y chromosome’s disease gene proportion is 82.61% higher, at 0.42. Meanwhile, the X chromosome’s proportion is 30.43% more than autosomes, sitting at 0.301. To understand if the differences between sex chromosomes and autosomes relate to reproductive functions, we divided disease genes into reproductive (1285 genes) and non-reproductive (3661 genes) categories based on affected organs (Supplementary tables 6 and 7). By fitting the number of disease genes against all dated genes on chromosomes, we observed that the X chromosome exhibited a bias towards reproductive functions. Specifically, on the X chromosome, disease genes affecting non-reproductive systems were slightly fewer than expected (−1.65% excess rate, with 154 observed versus 156.59 expected). The X chromosome displayed a significant surplus of reproductive-related disease genes (observed number 99, expected number 52.73, excess rate 87.75%, *p* < 5.56e-9) (Figure 3d).

Given the sex-imbalanced mode of inheritance for the X chromosome, theoretical models have predicted that purifying selection would remove both dominant female-detrimental mutations and recessive male-detrimental mutations (Rice 1984; Charlesworth et al. 1987). We determined that the ratio of male to female reproductive disease genes (M_disease_/F_disease_ or *α*_*d*_) is considerably higher for the X chromosome (80/9 = 8.89) than for autosomes on average (38/21 = 1.81, odds ratio = 16.08, 95% CI: 6.73–38.44, *p* < 0.0001). This suggests a disproportionate contribution of disease genes from the male hemizygous X chromosome compared to the female homozygous X. Thus, our analysis indicates that the abundance of disease genes on the X chromosome compared to autosomes might largely stem from male-specific functional effects. These data suggest that the overrepresentation of disease genes on the X chromosome primarily results from recessive X-linked inheritance affecting males, rather than dominant effects impacting both sexes.

### The genome-wide excess of male reproductive disease genes: the “faster-X” and “faster-male” effects.

To determine which gender (male or female) might influence the biased distribution of reproductive-related genes on different chromosomes, we focused on genes specific to male and female reproductive disease. Based on the HPO terms of abnormalities in reproductive organs and gene age dating, we retrieved 154 female-specific and 945 male-specific disease genes related to the reproductive system with age dating data (Supplementary Table 6 and 7). Through linear regression analysis, we assessed the number of gender-specific reproductive disease genes against the total counted genes for each chromosome. We observed strikingly different patterns that are dependent on gender and chromosomes.

For female reproductive disease genes, the X chromosome did not differ from autosomes, adhering to a linear autosomal pattern (*R*^2^ = 0.53, *p* = 1.04e-4, Figure 3e). However, for male reproductive disease genes, the X and Y chromosomes stood out compared to autosomes, which followed a linear pattern (*R*^2^ = 0.82, *p* = 5.56e-9, Figure 3f). The X chromosome contained 111.75% more male reproductive genes than expected. Moreover, compared to autosomes (averaging 38/853), the sex chromosomes, Y (17/45) and X (80/840), demonstrated significantly higher ratios of male reproductive disease genes, with odds ratios of 8.48 (95% CI: 4.45 – 16.17, *p* < 0.0001) and 2.14 (95% CI: 1.44 to 3.18, *p* = 0.0002), respectively. On the X chromosome, male reproductive genes outnumbered female ones by a factor of 10.43 (80/840 vs. 7/840). This observation is consistent with the “faster-X hypothesis”, where purifying selection is more effective in eliminating recessive deleterious mutations on the X chromosome due to the male hemizygosity of the X chromosome (Rice 1984; Charlesworth et al. 1987). Interestingly, a male bias was also observed in reproductive disease genes on autosomes, with the male linear model slope being approximately 4.21 times steeper than that for females (0.038 vs. 0.0073) (Figure 3e and 3f). Thus, our observed excess of male reproductive disease genes is not caused solely by the “faster-X” effect. It might also be influenced by the “faster-male” effect, postulating that the male reproductive system evolves rapidly due to heightened sexual selection pressures on males (Wu and Davis 1993).

### Excess of male reproductive disease genes at younger regions of X-chromosome.

**Figure 4.**
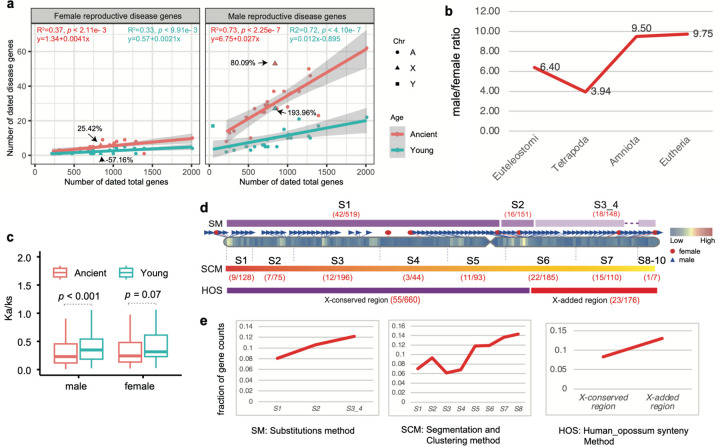
(**a**) Numbers of female-specific (left) and male-specific reproductive disease genes (right) are plotted against all protein-coding genes with gene ages on chromosomes. The linear formulas fitted for autosomal genes at ancient (Euteleostomi) and younger (post-Euteleostomi) stages are shown in red and blue, respectively. (**b**) The ratios of male to female reproductive disease gene numbers (α_d_) across four evolutionary epochs. (**c**) The comparison of selection pressure (human-chimpanzee pairwise Ka/Ks) for sex-specific reproductive disease genes between the ancient (stage Euteleostomi) and younger (post-Euteleostomi) epochs. Only the autosomal comparison is shown, with *p* value from the Wilcoxon test. (**d**) The numbers of male-specific reproductive disease genes (*m*) and the background genes (*b*) within the subregions from old to young on the X chromosome are provided, with the numbers displayed within round brackets for each subregion (*m*/*b*). SM, SCM, and HOS denote three classification methods for X chromosome structure: the substitutions method (SM), the segmentation and clustering method (SCM), and the synteny method (orthologous gene order conservation between human and opossum, HOS). (**e**) The fraction of disease genes with male-specific reproductive disease phenotypes within each stratum or subregion, as illustrated in (**d**), is presented. The gene coordinates have been updated based on the hg38 reference with liftover tool. “A”, “X”, and “Y” indicate autosomes, X and Y chromosomes, respectively.

While we observed a male-bias in reproductive disease genes, the influence of gene ages as a factor on this excess remains unclear. We compared gene distribution patterns between older (or ancient, stage Euteleostomi) and younger (post-Euteleostomi) stages. For female-specific reproductive disease genes, the X chromosome has an excess of ancient genes (25.42%) but a deficiency of young genes (57.16%) (Figure 4a). Conversely, for male-specific reproductive disease genes, younger genes exhibited a higher excess rate than ancient genes (193.96% vs. 80.09%) (Figure 4a). These patterns suggest an age-dependent functional divergence of genes on the X chromosome, which is consistent with gene expression data (Zhang et al. 2010). The X chromosome is “masculinized” with young, male-biased genes and old X chromosomal genes tend to be “feminized,” maintaining expression in females but losing it in males (Zhang et al. 2010). On autosomes, the linear regression slope values were higher for male reproductive disease genes than for female ones, both for ancient (0.027 vs. 0.0041) and young genes (0.012 vs. 0.0021) (Figure 4a). The ratio of male to female reproductive disease gene counts (*α*_*d*_) showed a predominantly male-biased trend across epochs, with a higher value in the most recent epoch of Eutheria (9.75) compared to the ancient epochs Euteleostomi and Tetrapoda (6.40 and 3.94, Figure 4b). Selection pressure comparison between young and ancient genes revealed no significant difference for female-specific reproductive disease genes, but significant difference for male-specific ones (Figure 4c, the Wilcoxon rank-sum test, *p* < 0.0001), indicating that young genes under male-biased sexual selection have less evolutionary constraints than older ones (median Ka/Ks ratios 0.35 vs. 0.23).

Structurally, the eutherian hemizygous X chromosome comprises an ancestral X-conserved region and a relatively new X-added region (Bellott et al. 2010). The ancestral X-conserved region is shared with the marsupial X chromosome, whereas the X-added region originates from autosomes (Figure 4d). To understand which human X chromosome regions might contribute differentially to human genetic disease phenotypes, we compared genes within the X-conserved and X-added regions, based on previous evolutionary strata and X chromosome studies (Ross et al. 2005; McLysaght 2008; Pandey et al. 2013). After excluding genes on X-PAR (pseudoautosomal regions) regions (Ensembl v110), we found that the proportion of male-specific reproductive disease genes in X-added region (13.07%, 23/176) exceeds that in the X-conserved region (8.33%, 55/660) (Figure 4d and 4e, Supplementary Table 8). Moreover, analyses of the evolutionary strata, which relies on substitutions method (Lahn and Page 1999a) and the segmentation and clustering method (Pandey et al. 2013), consistently showed higher fractions of male-specific reproductive disease genes in younger evolutionary strata than in older ones (Figure 4e). These observations indicate that, on the X chromosome, young genes could be more susceptible to male-biased sexual selection than old genes, despite their nearly identical hemizygous environment.

## Discussion

### The roles of young genes in human biomedically important phenotypes and innovations.

After the discovery of the first disease gene in 1983, which was based on linkage mapping for a Huntington’s disease with pedigree (Gusella et al. 1983), there has been a rapid advancement in medical genetics research. As of now, this field has identified approximately 20% of human genes (~4000–5000 genes) with phenotypes of the rare or “orphan” diseases (Asimit et al. 2012; Boycott et al. 2013; Henn et al. 2015; Krumm et al. 2015; Ji et al. 2016; Study 2017; Krausz and Riera-Escamilla 2018; Wen et al. 2018; Almlöf et al. 2019; Povysil et al. 2019; Thuresson et al. 2019; Guo et al. 2021; Oud et al. 2021). In our study, we utilized the latest disease gene and clinical phenotype data from HPO annotations (Köhler et al. 2018) and incorporated synteny-based gene age dating to account for new gene duplication events (Shao et al. 2019). Our synteny-based gene age dating reveals that younger genes have lower percentages of disease genes than older genes. This result is consistent with the pattern observed in the previous study (Domazet-Lošo and Tautz 2008). By utilizing DNVs and rare variants from large-scale datasets of exomic and genomic sequencing aggregated in recent years (Wang et al. 2022; Greene et al. 2023), we found that evolutionary older genes tend to have higher gene-wise DNVs burden. Logistic regression modeling indicates that protein length, gene age, and DNVs burden are positively correlated with the probability of genes as being disease genes. Thus, our finding suggests that the overrepresentation of disease genes in older evolutionary age groups could be due to the joint effect of deleterious variant burden, gene length, and gene age over evolutionary time under various forms of selection. Despite previous debates on the selective pressure of disease genes (Smith and Eyre-Walker 2003; Domazet-Lošo and Tautz 2008; Chakraborty et al. 2016; Spataro et al. 2017), our comparative analyses of Ka/Ks ratios between humans and primates consistently show stronger purifying selection on disease genes than non-disease genes, indicating evolutionary constraints to remove harmful mutations. The epoch-wise estimates of the emergence rate of disease genes per million years reveal a steady integration of genes into disease phenotypes, which supports Haldane’s seminal 1937 finding that new deleterious mutations are eliminated at the same rate they occur (Haldane 1937; Keightley 2012).

### Young genes rapidly acquire phenotypes under both sexual and natural selection.

The chromosomal distribution of all disease genes shows the excess of disease genes in X chromosome (Figure 3), which supports the “faster-X effect” (Rice 1984; Charlesworth et al. 1987), that male X-hemizygosity could immediately expose the deleterious X chromosome mutations to purifying selection. Conversely, the X-chromosome inactivation (XCI) in female cells could lessen the deleterious phenotypes of disease variants on the X chromosome (Migeon 2020). The X chromosome excess of disease genes is attributed predominantly to that of the male reproductive disease genes (Figure 3). This male-specific bias was not limited to the sex chromosome but also detectable in autosomes (Figure 3). These findings align with the “faster-male” effect, where the reproductive system evolves more rapidly in males than in females due to heightened male-specific sexual selection (Wu and Davis 1993). Intriguingly, of the 22 HPO systems, young genes are enriched in disease phenotypes affecting the reproductive-related system. As genes age, there’s a marked decline in both PEI (phenotype enrichment index) and (the male-to-female ratio of reproductive disease gene numbers). These patterns are consistent with the “out of testis” hypothesis (Kaessmann 2010), which describes the male germline as a birthplace of new genes due to factors including the permissive chromatin state and the immune environment in testis (Vinckenbosch et al. 2006; Bekpen et al. 2018). The “out of testis” hypothesis predicts that genes could gain broader expression patterns and higher phenotypic complexity over evolutionary time (Vinckenbosch et al. 2006). Consistently, we observed a pattern where older sets of disease genes have phenotypes over a much broader anatomical systems compared to younger genes which tend to impact limited systems. The strong enrichment of male reproductive phenotypes for young genes is also consistent with findings from model species that new genes often exhibit male-reproductive functions (Betrán et al. 2002; Heinen et al. 2009), in both *Drosophila* (Heinen et al. 2009; Gubala et al. 2017; VanKuren and Long 2018) and mammals (Emerson et al. 2004; Jiang et al. 2017). Some new gene duplicates on autosomes are indispensable during male spermatogenesis, to preserve male-specific functions that would otherwise be silenced on the X chromosome due to the meiotic sex chromosome inactivation (MSCI) (Emerson et al. 2004; Zhang et al. 2010; Jiang et al. 2017).

Apart from the reproductive functions, new genes are also enriched for adaptive phenotypes. Previous transcriptomic studies indicate that new genes have excessive upregulation in the human neocortex and under positive selection (Zhang et al. 2011). The brain size enlargement, especially the neocortex expansion over ~50% the volume of the human brain, ranks among the most extraordinary human phenotypic innovations (Rakic 2009; Zhang et al. 2011). Here, we found that at least 42% of primate-specific disease genes affecting the nervous systems could impact phenotypes related to brain size and intellectual development. For example, *DDX11* is critical in pathology of microcephaly (Pirozzi et al. 2018; Lerner et al. 2020; van Schie et al. 2020; Ma et al. 2022). The *NOTCH2NLA*, *NOTCH2NLB,* and *NOTCH2NLC* may promote human brain size enlargement, due to their functions in neuronal intranuclear inclusion disease (NIID), microcephaly, and macrocephaly (Fiddes et al. 2018; Suzuki et al. 2018; Liu et al. 2022). The *RRP7A* is also a microcephaly disease gene evidenced from patient-derived cells with defects in cell cycle progression and primary cilia resorption (Farooq et al. 2020). The defects of *SMPD4* can lead to a neurodevelopmental disorder characterized by microcephaly and structural brain anomalies (Magini et al. 2019). The *SRGAP2C* accounts for human-specific feature of neoteny and can promote motor and execution skills in mouse and monkey model (Charrier et al. 2012; Dennis et al. 2012; Meng et al. 2023). The *de novo* gene *SMIM45* (Delihas 2023) associates with cortical expansion based on extensive models (An et al. 2023).

New genes were also found with enrichment in other adaptive phenotypes, particularly involving the head and neck, eye, and musculoskeletal system. Some examples of these primate-specific disease genes encompass *CFHR3* associated with macular degeneration (Fritsche et al. 2016), *SMPD4* with the retinopathy (Smits et al. 2023), *TUBA3D* with the keratoconus (Hao et al. 2017), *OPN1MW* with loss of color vision (Winderickx et al. 1992; Ueyama et al. 2002), *YY1AP1* with Fibromuscular dysplasia (Guo et al. 2017), *SMN2* with the Spinal Muscular Atrophy (Hahnen et al. 1996), *GH1* with defects in adult bone mass and bone loss (Dennison et al. 2004), *KCNJ18* with thyrotoxicosis complicated by paraplegia and hyporeflexia (Ryan et al. 2010), *TBX5* with the cardiac and limb defects of Holt-Oram syndrome (Basson et al. 1997; Li et al. 1997), and *DUX4* with muscular dystrophy (Lemmers et al. 2012). Additionally, some other specific functions have also been reported for these young genes. For example, the Y chromosome gene *TBL1Y* could lead to male-specific hearing loss (Di Stazio et al. 2019). The *TUBB8* defects could lead to complete cleavage failure in fertilized eggs and oocyte maturation arrest (Feng et al. 2016; Yuan et al. 2018; Yao et al. 2022). Interestingly, a previous case study on mice also shows the role of *de novo* genes on female-specific reproductive functions (Xie et al. 2019). These emerging studies independently support the importance of new genes in phenotypic innovation and sexual selection, refuting previous assumptions that new genes contribute little to phenotypic innovation (Carroll 1995).

### New genes underlying rapid phenotypic innovations: low pleiotropy as a selective advantage.

**Figure 5.**
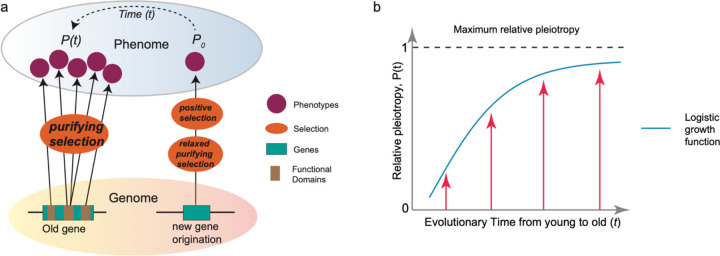
(**a**) The ‘pleiotropy-barrier model’ posits that new genes evolve adaptively more quickly. It suggests that older genes undergo stronger purifying selection because their multiple functions (usually adverse pleiotropy) act as a barrier to the uptake of mutations that might otherwise be beneficial for novel phenotypes. (**b**) The logistic function between relative pleiotropy P(*t*) and evolutionary time *t*, $P\text{(}t\text{)}=\frac{P\_max}{1+e^{-kt}}$, where *P_max* represents the maximum relative pleiotropy. The *k* is the growth rate parameter, which controls how quickly the phenomenon approaches the maximum value. A higher k value means faster growth initially.

Our findings raise the question of why new genes can quickly enrich into phenotypic traits that are crucial for both sexual evolution and adaptive innovation. This question could not be fully addressed by previous hypotheses. The “out of testis” theory, as well as the “male-driven,” “faster-X,” and “faster-male” theories, do not offer specific predictions regarding the propensity of new or young genes to be involved in adaptive traits. Here, we proposed a “pleiotropy-barrier” hypothesis to explain the relationship between innovation potential and gene ages (Figure 5a). The recognition of pleiotropy is as early as biology itself as a research field (Mendel 1866; Wright 1984; Barton 1990; Baatz and Wagner 1997). Mendel’s classic paper in 1866 suggests a single factor controls three characters of Pisum (Mendel 1866). Before Mendel, many medical syndromes had already described syndromes with different symptoms and a single “familial” factor (Eckman 1788). It is established that young genes exhibit higher specificity and narrower expression breadth across tissues (Zhang et al. 2012). In this study, we used a broader definition of pleiotropy to understand phenotype evolution (Pyeritz 1989; Lobo 2008; Tyler et al. 2009; Zhang 2023). We reveal a pattern that older genes tend to impact more organs/systems, while young genes display phenotype enrichment in specific organs (Figure 2c). Therefore, both phenotype pattern and expression trend across evolutionary epochs suggest lower pleiotropy for young genes, compared to the progressively higher pleiotropy observed in older genes.

Numerous theoretical and genomic studies have revealed that pleiotropy impedes evolutionary adaptation (a so-called ‘cost of complexity’) (Zeng and Hill 1986; Baatz and Wagner 1997; Orr 2000; Wagner and Zhang 2011; Fraïsse et al. 2018; Quiver and Lachance 2022), while low pleiotropy could foster morphological evolution (Carroll 2005; Wray 2007). The inhibitory effect of pleiotropy on novel adaptation aligns with our observations of the strong purifying selection on both high extent of pleiotropy (Wagner and Zhang 2011; Quiver and Lachance 2022) and expression breadth (Zhu et al. 2008). As expected, we observed that multi-system genes and older genes, which exhibit higher pleiotropy, undergo stronger purifying selection (Figure 1g-1h). This evolutionary constraint suggests a restricted mutation space to introduce novel traits for old genes due to the “competing interests” of multifunctionality (Figure 5). The inhibitory pressure could also reduce genetic diversity due to background selection (Charlesworth et al. 1993). The evolution of new genes, especially gene duplicates, serves as a primary mechanism to mitigate pleiotropic effects through subfunctionalization and neofunctionalization (He and Zhang 2005; Guillaume and Otto 2012) and avoid adverse pleiotropy in ancestral copies (Hoekstra and Coyne 2007). The tissue-specific functions of new genes, as a general pattern in numerous organisms, could circumvent the adaptive conflicts caused by the multifunctionality of parental genes (Des Marais and Rausher 2008). The reduced pleiotropy in young genes could thereby allow for a more diverse mutational space for functional innovation without triggering unintended pleiotropic trade-offs (Dezső et al. 2008).

The “pleiotropy-barrier” model predicts that the capacity for phenotypic innovation is limited by genetic pleiotropy under nature selection (Figure 5a). Over evolutionary time, the pleiotropy increase follows a logistic growth pattern, where the speed of growth could be higher for younger genes but lower for older genes (Figure 5b). The multifunctional genes could encounter an escalating “barrier” toward the pleiotropy maximum. This barrier arises because more functions necessitate stronger selective constraints, which could in turn reduce mutational space of beneficial mutations for novel phenotypes. In contrast, low or absent pleiotropy in new genes allows for a higher and tunable mutation space under the relaxed purifying selection. The permissive environment provides a fertile ground for beneficial mutations to appear with novel functions. Such innovations, initially as polymorphisms within a population, can become advantageous phenotypes and ready responder in certain environment under positive selection. Notably, high pleiotropy could also hamper the resolution of sexual conflict through the sex-limited expression (Judith E. Mank et al. 2008). Thus, the “pleiotropy-barrier” model also applies to the evolution of new genes with male-specific functions under sexual selection (VanKuren and Long 2018). Therefore, new or young genes, with lower pleiotropic effect as a selective advantage, not only spurs molecular evolution under sexual and natural selection but, from a medical standpoint, also are promising targets for precise medicine, warranting deeper investigation.

## Conclusion

By combining human gene age dating and Mendelian disease phenotyping, we revealed an increase trend of disease gene proportions over evolutionary time. This growth pattern could be due to higher gene-wise deleterious variant burdens in older genes. We found that the ratio of genes associated with health-related functions per million years remains relatively consistent across macroevolutionary epochs. Importantly, young genes are preferentially linked to disease phenotypes of the male reproductive system, as well as systems that undergone significant phenotypic innovations in primate or human evolution, including the nervous system, head and neck, eyes, and the musculoskeletal system. The enrichment of these disease systems points to the driving forces of both sexual selection and adaptive evolution for young genes. As evolutionary time progresses, older genes display fewer specialized functions compared to their young counterparts. Our findings highlight that young genes are likely the frontrunners of molecular evolution, being actively selected for functional roles by both adaptive innovation and sexual selection, a process aided by their lower pleiotropy. Therefore, young genes play a pivotal role in addressing a multitude of questions related to the fundamental biology of humans.

## Materials and Methods

### Gene age dating and disease phenotypes

The gene age dating was conducted using an inclusive approach. For autosomal and X chromosomal genes, we primarily obtained gene ages (or branches, origination stages) from the GenTree database (Zhang et al. 2010; Shao et al. 2019) that is based on Ensembl v95 of human reference genome version hg38 (Flicek et al. 2014). We then trans-mapped the v95 gene list of GenTree into the current release of Ensembl gene annotation (v110). The gene age inference in the GenTree database relied on genome-wide synteny and was based on the presence of syntenic blocks obtained from whole-genome alignments between human and outgroup genomes (Zhang et al. 2010; Long et al. 2013; Shao et al. 2019). The most phylogenetically distant branch at which the shared syntenic block was detected marks the time when a human gene originated. In comparison to the method based on the similarity of protein families, namely the phylostratigraphic dating (Neme and Tautz 2013), this method employed in GenTree is robust to recent gene duplications (Shao et al. 2019), despite its under-estimation of the number of young genes (Ma et al. 2022). We obtained gene age for human Y genes through the analysis of 15 representative mammals (Cortez et al. 2014). Notably, Y gene ages are defined as the time when these genes began to evolve independently of their X counterpart or when they translocated from other chromosomes to the Y chromosome due to gene traffic (transposition/translocation) (Cortez et al. 2014). For the remaining Ensembl v110 genes lacking age information, we dated them using the synteny-based method with the gene order information from ENSEMBL database (v110), following the inference framework of GenTree (Shao et al. 2019). These comprehensive methods resulted in the categorization of 19,665 protein-coding genes into distinct gene age groups, encompassing evolutionary stages from Euteleostomi to the human lineage, following the phylogenetic framework of the GenTree database. The HPO annotation used in this study for phenotypic abnormalities contains disease genes corresponding to 23 major organ/tissue systems (09/19/2023, https://hpo.jax.org/app/data/annotations). After filtering out mitochondrial genes, unplaced genes, RNA genes, and genes related to neoplasm ontology, we obtained with gene ages and phenotypic abnormalities (across 22 categories) for 4946 protein-coding genes. The reproductive system disease genes were retrieved from the “phenotype to genes.txt” file based on “reproduct”, “male”, “female” keywords (neoplasm-related items were removed).

### Logistic regression modeling and model comparison

We retrieved the burden of de novo germline variants from multiple studies, including the Gene4Denovo database (68,404 individuals) (Zhao et al. 2020), UK Biobank exomes (394,783 individuals), and gnomAD database (V4.0, 76,215 individuals) (Wang et al. 2022; Greene et al. 2023). The genotypes and allele frequencies were downloaded from gnomAD (https://gnomad.broadinstitute.org/downloads). Rare variants were extracted based on MAF lower than 0.0001 across all major human populations (all human male, all human female, African population, non-Finish European population, East Asian population, South Asian population, and Latino/Admixed American population). We conducted the stratified Logistic regression to account for the effects of multiple predictors and their interactions on the outcome of gene disease states. The disease and non-disease genes were assigned into binary states (“1”, disease genes; “0”, non-disease genes) as response variable. A step-by-step procedure was performed for multiple predictors, which include gene age (*T, mya*), gene length (*L*_*g*_) or protein length (*L*), DNVs burden (*D*) (Wang et al. 2022), and rare variant burden (*R*) (Chen et al. 2024b). The likelihood ratio test (LRT) and Akaike information criterion (AIC) were used for model comparison (Table 1). Lower AIC was preferred if the same degree of freedom was detected. The model with significant LRT *p*-value (*p* < 0.05) was chosen when comparing nested models. To account for different scales in variables, the models with logarithm treatment were also incorporated and compared based on AIC criteria. The Variance Inflation Factor (VIF) values were used to account for the multicollinearity among variables. The “glm” package (binomial model) in R platform was used for computing the models (https://www.rdocumentation.org/packages/stats/versions/3.6.2/topics/glm).

### Ka/Ks ratio

Ka/Ks is widely used in evolutionary genetics to estimate the relative strength of purifying selection (Ka/Ks < 1), neutral mutations (Ka/Ks = 1), and beneficial mutations (Ka/Ks > 1) on homologous protein-coding genes. Ka is the number of nonsynonymous substitutions per non-synonymous site, while Ks is the number of synonymous substitutions per synonymous site that is assumed to be neutral. The pairwise Ka/Ks ratios (human-chimpanzee, human-bonobo, and human-macaque) were retrieved from the Ensembl database (v99) (Flicek et al. 2014), as estimated with the Maximum Likelihood algorithm (Yang 2007).

### Disease gene emergence rate per million years (r)

To understand the origination tempo of disease genes within different evolutionary epochs, we estimated the disease gene emergence rate per million years *r* for disease genes, which is the fractions of disease genes per million years for each evolutionary branch. The calculating is based on the following formula:

$$r_{i}=\frac{O_{i}}{A_{i}T_{i}}$$

where $r_{i}$ represents the phenotype integration index for ancestral branch i. The $O_{i}$ indicates the number of disease genes with organ phenotypes in ancestral branch i. The denominator $A_{i}$ is the number of genes with gene age information in branch i. The $T_{i}$ represents the time obtained from the Timetree database (http://www.timetree.org/) (Kumar et al. 2017).

### Pleiotropic modeling with logistic growth function

For each evolutionary epoch (t), we estimated the median numbers of OP systems that genic defects could affect, which serve as the proxy of pleiotropy over evolutionary time (P(t)) for regression analysis. The logistic growth function was used to fit the correlation with the Nonlinear Least Squares in R.

### Phenotype enrichment along evolutionary stages

The phenotype enrichment along evolutionary epochs was evaluated based on a phenotype enrichment index (PEI). Specifically, within “gene-phenotype” links, there are two types of contributions for a phenotype, which are “one gene, many phenotypes” due to potential pleiotropism as well as “one gene, one phenotype”. Considering the weighting differences between these two categories, we estimated the $PEI_{\left(i,j\right)}$ for a given phenotype $\left(p_{i}\right)$ within an evolutionary stage $\left(br_{j}\right)$ with the following formula.


$${\text{PEI}}_{\left(i,j\right)}=\frac{\sum_{i=1}^{n}\frac{1}{m_{i}}}{\sum_{J=1}^{l}\sum_{k=1}^{n_{j}}\frac{1}{m_{k}}}$$

The m indicates the number of phenotype(s) one gene can affect, n represents the number of genes identified for a given phenotypes, and l is number of phenotypes within a given evolutionary stage. Considering the genetic complexity of phenotypes, the enrichment index (*PEI*) firstly adjusted the weights of genes related to a phenotype with the reciprocal value of m, *i.e*., $\frac{1}{m}$. Thus, the more phenotypes a gene affects, the less contributing weight this gene has. Here, $m_{i}$ is the number of phenotypes affected by the i-th gene, n is the total number of genes associated with the specific phenotype $p_{i}$, $n_{j}$ is the number of genes associated with the j-th phenotype within the evolutionary stage, and $m_{k}$ is the number of phenotypes affected by the k-th gene within the j-th phenotype. Then, we can obtain the accumulative value $\left(p\right)$ of the adjusted weights of all genes for a specific phenotype within an evolutionary stage. Because of the involvement of multiple phenotypes within an evolutionary stage, we summed weight values for all phenotypes $\left(\sum_{J=1}^{k}p\right)$ and finally obtained the percentage of each phenotype within each stage $\left(\frac{p}{\sum_{J=1}^{l}p}\right)$ as the enrichment index.

### The linear regression and excessive rate

The linear regression for disease genes and total genes on chromosomes was based on the simple hypothesis that the number of disease genes would be dependent on the number of total genes on chromosomes. The linear regression and statistics were analyzed with R platform. The excessive rate was calculated as the percentages (%) of the vertical difference between a specific data point, which is the number of gene within a chromosome (*n*), and the expected value based on linear model (*n-e*) out of the expected value $\left(\frac{n-e}{e}\right)$.

### The X-conserved and X-added regions

The Eutherian X chromosome is comprised of the pseudoautosomal regions (PAR), X-conserved region, and X-added region. The regions of two PAR were determined based on NCBI assembly annotation of GRCh38.p13 (X:155701383–156030895 and X:10001–2781479). The X-boundary between X-conserved and X-added regions was determined with Ensembl biomart tool. The “one to one” orthologous genes between human and opossum were used for gene synteny identification. The X-conserved region is shared between human and opossum, while X-added region in human has synteny with the autosomal genes of opossum (Ross et al. 2005). The “evolutionary strata” on X were based on previous reports of two methods: substitutions method and the Segmentation and Clustering method (Lahn and Page 1999b; McLysaght 2008; Pandey et al. 2013). The coordinates of strata boundaries were up-lifted into hg38 genome with liftover tool (https://genome.ucsc.edu/cgi-bin/hgLiftOver).

## Supplementary Material

Supplement 1

Supplement 2

## Data Availability

The gene age dating and disease gene phenotyping data were listed in the Supplementary Table. Other important data, including the files of rare variants and allele frequencies across global human populations (76,215 individuals) and the R script for modeling, could be retrieved from Zenodo (https://zenodo.org/uploads/11000269).
