# Supplementary material for: Evolutionarily new genes in humans with disease phenotypes reveal functional enrichment patterns shaped by adaptive innovation and sexual selection
Source: bioRxiv. 2024 Sep 4:2023.11.14.567139. Preprint. [Version 7] doi: 10.1101/2023.11.14.567139 (PMC10690195; doi:10.1101/2023.11.14.567139)
Supplement: Supplement 2 [file media-2.pdf]

Supplemental Figure S2.

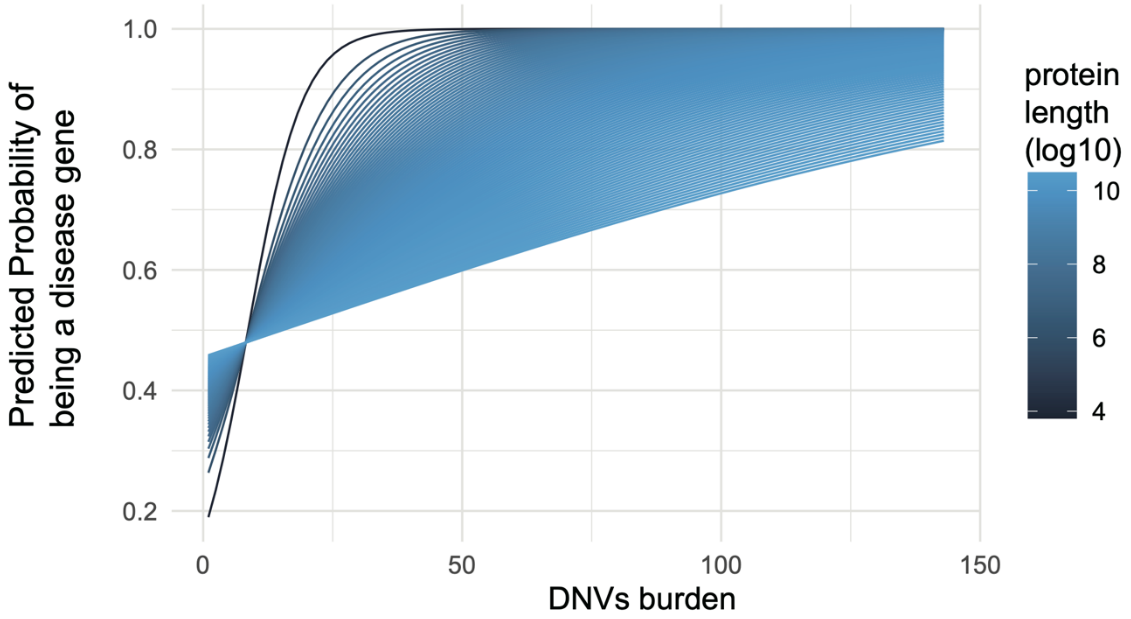

The interaction plot between DNVs burden and protein length (at logarithm scale) for the predicted probability of being a disease gene. The details of full model are shown in Supplemental Table 4.
