# Supplementary material for: Evolutionarily new genes in humans with disease phenotypes reveal functional enrichment patterns shaped by adaptive innovation and sexual selection
Source: bioRxiv. 2024 Sep 4:2023.11.14.567139. Preprint. [Version 7] doi: 10.1101/2023.11.14.567139 (PMC10690195; doi:10.1101/2023.11.14.567139)
Supplement: Supplement 3 [file media-3.pdf]

Supplemental Figure S3.

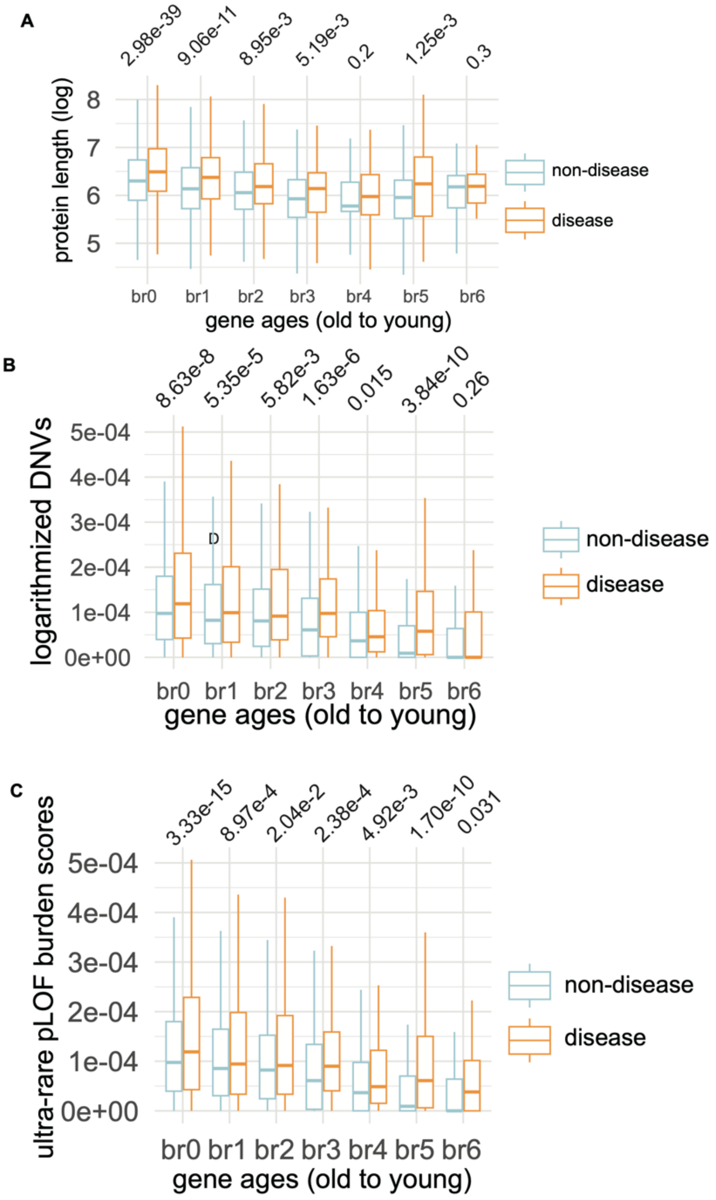

The relationship between multiple features (protein length, burden of DNVs, ultra-rare pLOF burden score) and seven gene age groups (phylostrata). (*A*) The comparison of protein lengths across gene ages between disease genes and non-disease genes. (*B*) The comparison of DNVs burdens across gene ages between disease genes and non-disease genes. (*C*) The comparison of ultra-rare pLOF burden scores across gene ages between disease genes and non-disease genes.
