# Supplementary material for: Evolutionarily new genes in humans with disease phenotypes reveal functional enrichment patterns shaped by adaptive innovation and sexual selection
Source: bioRxiv. 2024 Sep 4:2023.11.14.567139. Preprint. [Version 7] doi: 10.1101/2023.11.14.567139 (PMC10690195; doi:10.1101/2023.11.14.567139)
Supplement: Supplement 4 [file media-4.pdf]

Supplemental Figure S4.

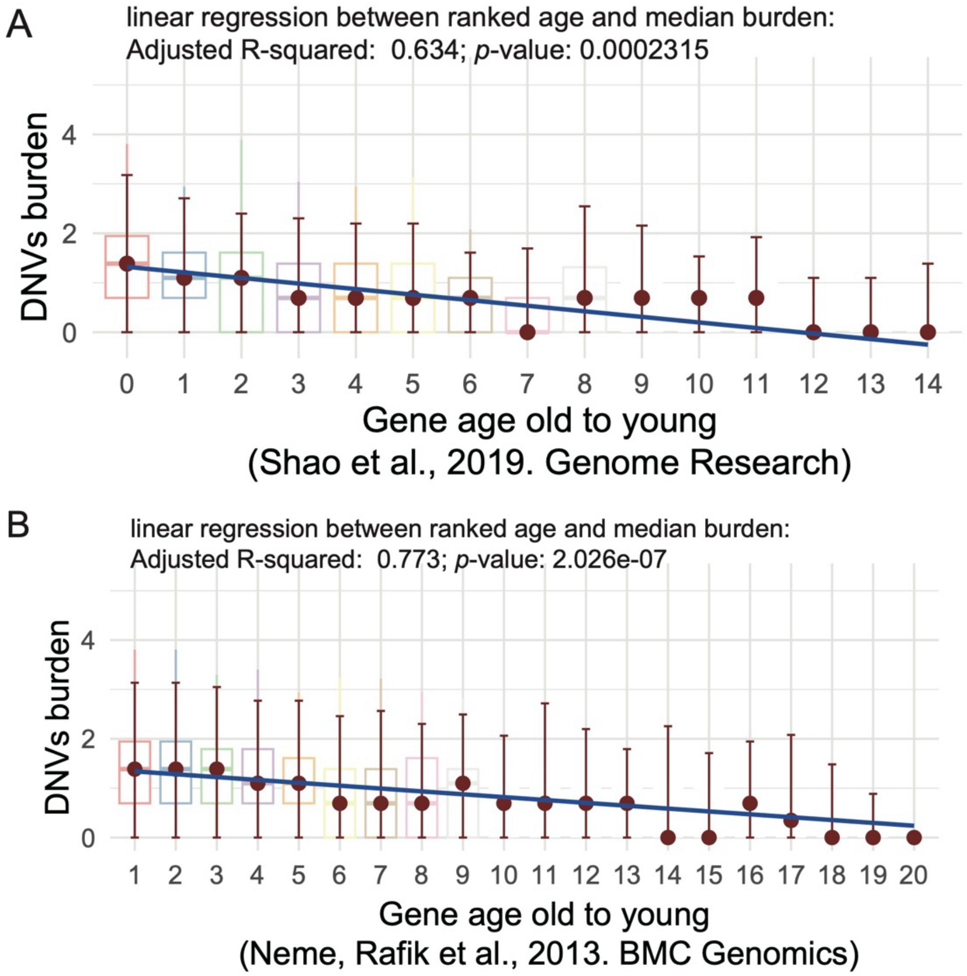

The relationship between two types of gene age dating and the DNVs burden. (*A*)The relationship between gene-wise DNVs burden from 68,404 individuals (Zhao et al. 2020) and the previously reported synteny-based gene age (Shao et al. 2019). (*B*) The relationship between gene-wise DNVs burden from 68,404 individuals (Zhao et al. 2020)and gene-family based gene age (Neme and Tautz 2013). Note: the linear models are between median values and ranked ages. The significance *p* values are shown above age groups (the one-tail Wilcoxon rank sum test with continuity correction).
