# Supplementary material for: Evolutionarily new genes in humans with disease phenotypes reveal functional enrichment patterns shaped by adaptive innovation and sexual selection
Source: bioRxiv. 2024 Sep 4:2023.11.14.567139. Preprint. [Version 7] doi: 10.1101/2023.11.14.567139 (PMC10690195; doi:10.1101/2023.11.14.567139)
Supplement: Supplement 5 [file media-5.pdf]

Supplemental Figure S5.

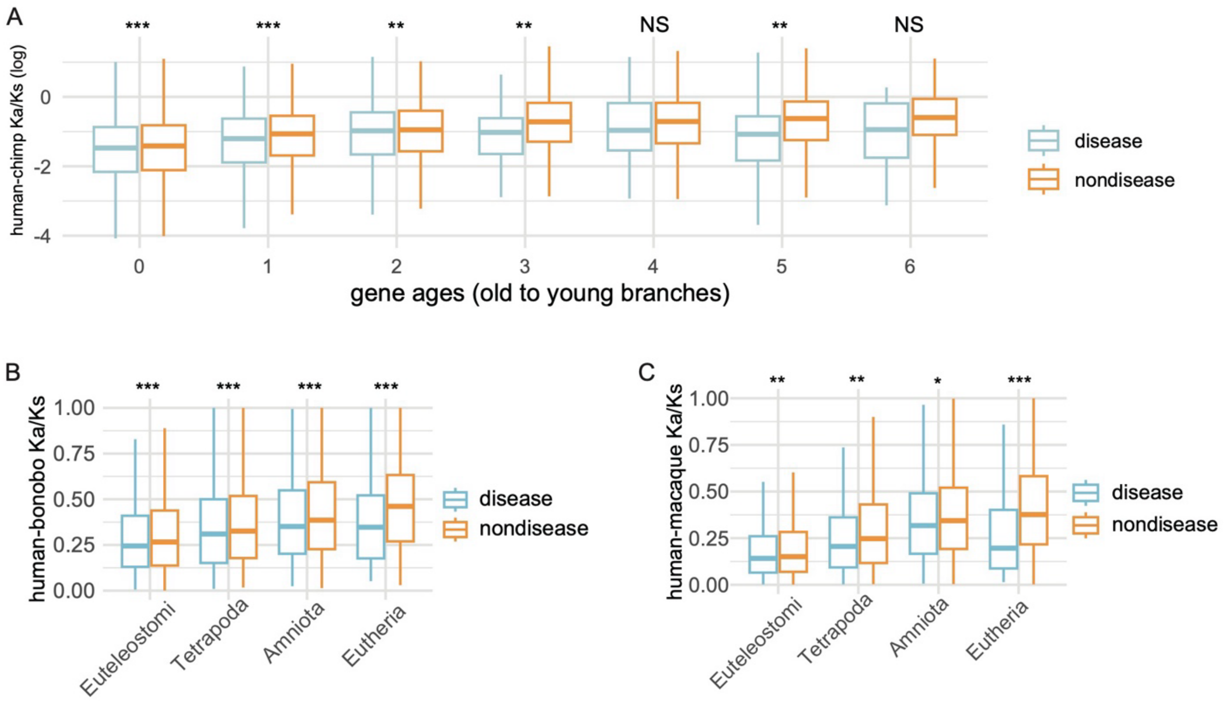

The pairwise Ka/Ks ratios from the Ensembl database based on the Maximum Likelihood estimation for "one-to-one" orthologs between human and other species. (*A*) The pairwise Ka/Ks ratios between human and chimpanzee across seven age groups. (*B*) The pairwise Ka/Ks ratios between human and bonobo across four age groups. (*C*) The pairwise Ka/Ks ratios between human and macaque across four age groups. Only genes under purifying selection are visualized (Ka/Ks < 1). Note: significance levels are based on the Wilcoxon rank sum test comparing disease genes and non-disease genes (one tail test). "*", "**", "***" indicate $p < 0.05$, < 0.01, < 0.001, respectively.
