# Supplementary material for: Evolutionarily new genes in humans with disease phenotypes reveal functional enrichment patterns shaped by adaptive innovation and sexual selection
Source: bioRxiv. 2024 Sep 4:2023.11.14.567139. Preprint. [Version 7] doi: 10.1101/2023.11.14.567139 (PMC10690195; doi:10.1101/2023.11.14.567139)
Supplement: Supplement 7 [file media-7.pdf]

Supplemental Figure S7.

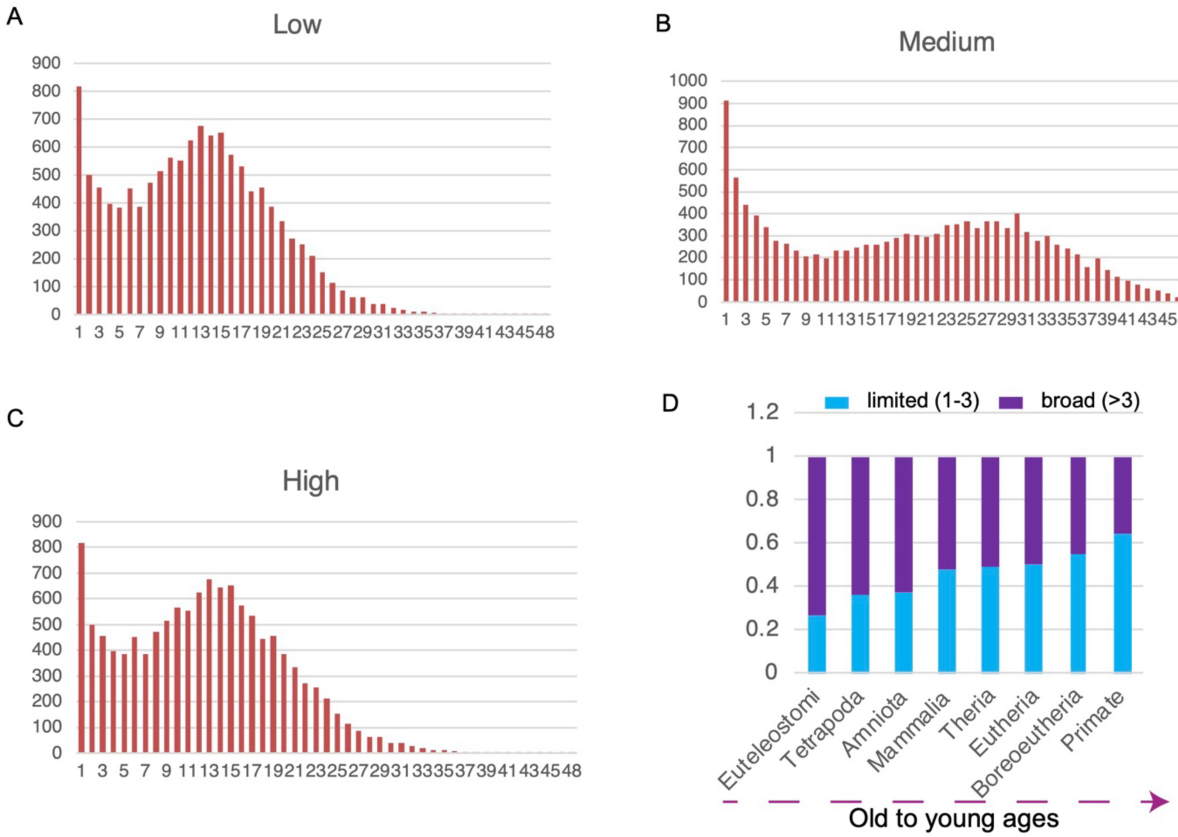

The distribution of gene expression breadth (the number of tissues) with RNASEQ data (The Human Protein Atlas, or HPA, normal tissues). (*A*) The distribution of counts of normal tissues with low levels of gene expression (based on HPA annotation). (*B*) The distribution of counts of normal tissues with medium levels of gene expression (based on HPA annotation). (*C*) The distribution of counts of normal tissues with high levels of gene expression (HPA annotation). (*D*) The distribution percentages of genes expressed in multiple tissues (>3) compared to those expressed in a limited number of tissues (<=3) across different evolutionary ages (only “HIGH” expression genes based on HPA are used).
