# Supplementary material for: Evolutionarily new genes in humans with disease phenotypes reveal functional enrichment patterns shaped by adaptive innovation and sexual selection
Source: bioRxiv. 2024 Apr 22:2023.11.14.567139. Preprint. [Version 6] doi: 10.1101/2023.11.14.567139 (PMC10690195; doi:10.1101/2023.11.14.567139)
Supplement: Supplementary file 1 [file NIHPP2023.11.14.567139v6-supplement-2.pdf]

bioRxiv preprint doi: https://doi.org/10.1101/2023.11.14.567139; this version posted April 22, 2024. The copyright holder for this preprint (which was not certified by peer review) is the author/funder, who has granted bioRxiv a license to display the preprint in perpetuity. It is made available under aCC-BY 4.0 International license.

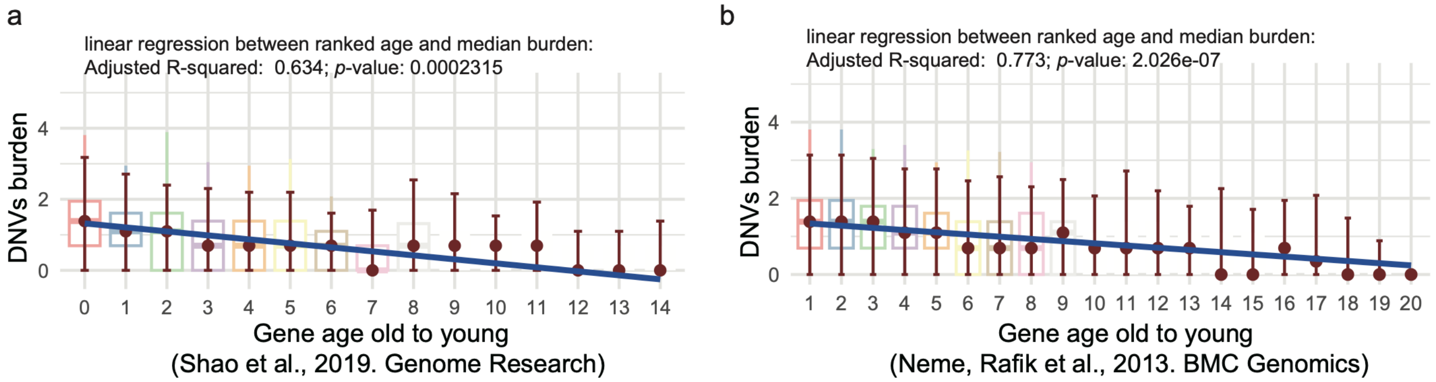

Supplementary Figure 1. The relationship between gene-wise DNVs burden from 68,404 individuals (Zhao et al. 2020) and the previously reported synteny-based gene age (Shao et al. 2019) (**a**) and gene-family based gene age (Neme and Tautz 2013) (**b**). Note: the linear models are between median values and ranked ages.

bioRxiv preprint doi: https://doi.org/10.1101/2023.11.14.567139; this version posted April 22, 2024. The copyright holder for this preprint (which was not certified by peer review) is the author/funder, who has granted bioRxiv a license to display the preprint in perpetuity. It is made available under aCC-BY 4.0 International license.

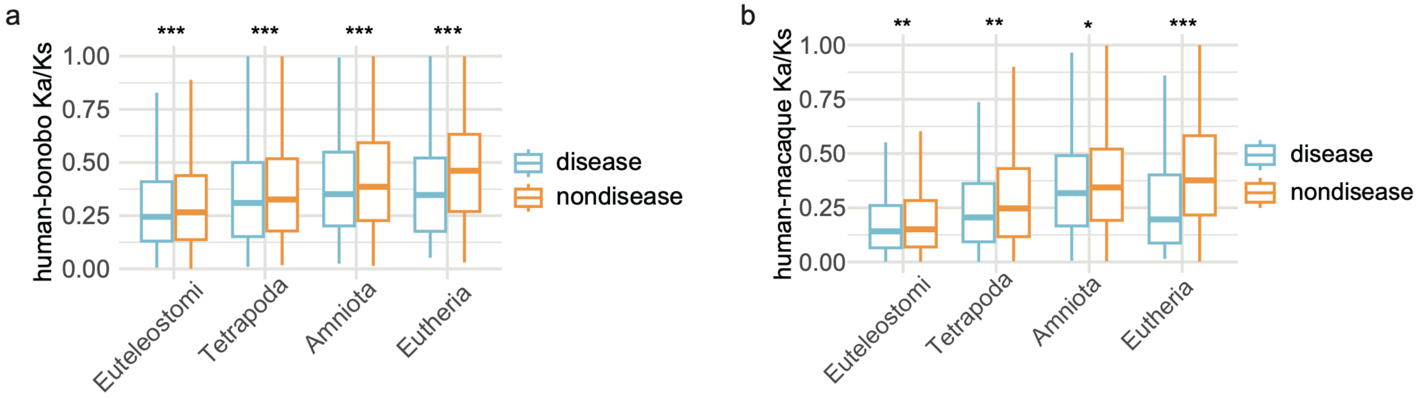

Supplementary Figure 2. The pairwise Ka/Ks ratios from Ensembl database based on the Maximum Likelihood estimation for "one to one" orthologs between human and bonobo (**a**) and between human and macaque (**b**). Only genes under purifying selection are visualized (Ka/Ks < 1). Note: the significance levels are based on the Wilcoxon rank sum test comparing disease genes and non-disease genes (one tail test). "*", "**", "***" indicate $p < 0.05$, < 0.01, < 0.001, respectively.

bioRxiv preprint doi: https://doi.org/10.1101/2023.11.14.567139; this version posted April 22, 2024. The copyright holder for this preprint (which was not certified by peer review) is the author/funder, who has granted bioRxiv a license to display the preprint in perpetuity. It is made available under aCC-BY 4.0 International license.

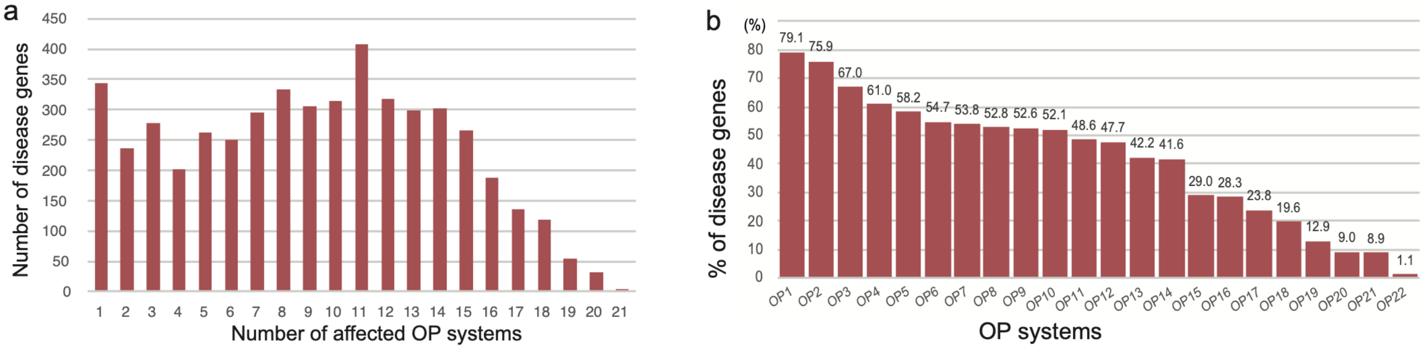

Supplementary Figure 3. The distributions of disease genes across the affected OP systems. (**a**) The distribution of OP system numbers on which disease gene defects could affect. The horizontal axis is the numbers of affected OP systems, and the vertical axis is the number of genes. (**b**) The percentages of disease genes out of all disease genes for different OP systems (OP1 to OP22). The definition of OP systems is consistent with Figure 1b.

bioRxiv preprint doi: https://doi.org/10.1101/2023.11.14.567139; this version posted April 22, 2024. The copyright holder for this preprint (which was not certified by peer review) is the author/funder, who has granted bioRxiv a license to display the preprint in perpetuity. It is made available under aCC-BY 4.0 International license.

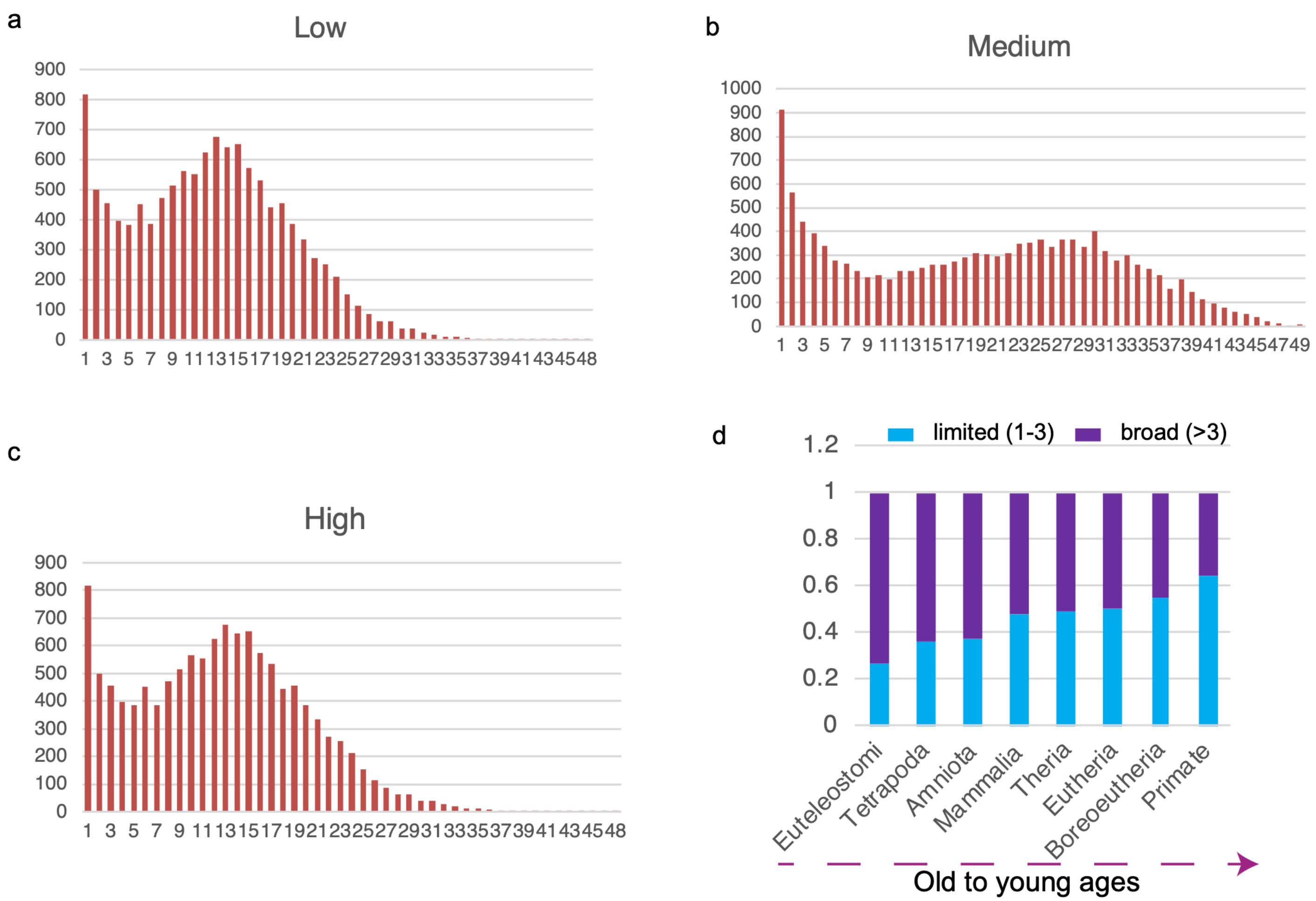

Supplementary Figure 4. The distribution for the numbers of tissues with RNASEQ expression (HPA normal tissues). (**a**) The distribution of counts of normal tissues with low levels of gene expression (HPA annotation). (**b**) The distribution of counts of normal tissues with medium levels of gene expression (HPA annotation). (**c**) The distribution of counts of normal tissues with high levels of gene expression (HPA annotation). (**d**) The distribution percentages of genes expressed in multiple tissues (>3) compared to those expressed in a limited number of tissues (<=3) across different evolutionary ages (only “HIGH” expression genes based on HPA are used).
